# Exploring the impact of trauma type and extent of exposure on posttraumatic alterations in 5-HT1A expression

**DOI:** 10.1038/s41398-020-00915-1

**Published:** 2020-07-16

**Authors:** Michael W. Lewis, Russell T. Jones, Margaret T. Davis

**Affiliations:** 1 Virginia Tech, Blacksburg, VA USA; 2 Yale University, New Haven, CT USA

**Keywords:** Pathogenesis, Molecular neuroscience

## Abstract

The long-term behavioral, psychological, and neurobiological effects of exposure to potentially traumatic events vary within the human population. Studies conducted on trauma-exposed human subjects suggest that differences in trauma type and extent of exposure combine to affect development, maintenance, and treatment of a variety of psychiatric syndromes. The serotonin 1-A receptor (5-HT1A) is an inhibitory G protein-coupled serotonin receptor encoded by the *HTR1A* gene that plays a role in regulating serotonin release, physiological stress responding, and emotional behavior. Studies from the preclinical and human literature suggest that dysfunctional expression of 5-HT1A is associated with a multitude of psychiatric symptoms commonly seen in trauma-exposed individuals. Here, we synthesize the literature, including numerous preclinical studies, examining differences in alterations in 5-HT1A expression following trauma exposure. Collectively, these findings suggest that the impact of trauma exposure on 5-HT1A expression is dependent, in part, on trauma type and extent of exposure. Furthermore, preclinical and human studies suggest that this observation likely applies to additional molecular targets and may help explain variation in trauma-induced changes in behavior and treatment responsivity. In order to understand the neurobiological impact of trauma, including the impact on 5-HT1A expression, it is crucial to consider both trauma type and extent of exposure.

## Introduction

Exposure to potentially traumatic events is common, with an estimated rate of over 80% in the United States and 70% worldwide^1^. However, long-term behavioral, psychological, and neurobiological effects of exposure vary considerably^2^. Some individuals experience rapid and sustained natural recovery, while others develop chronic trauma-related psychopathology^3^. Importantly, the nature and extent of trauma exposure combine to produce different outcomes; research suggests that exposure to qualitatively different events and different degrees of exposure lead to different psychiatric and neurobiological outcomes^4–21^. For example, qualitatively different events (e.g., rape, assault, and natural disaster) are associated with different levels of conditional risk for posttraumatic stress disorder (PTSD; 19% rape and 0.3% natural disaster)^20^. Similarly, different trauma types are differentially correlated with the emergence of several other psychiatric diagnoses and sequalae following trauma exposure (e.g., depression, anxiety, substance abuse, conduct problems, eating disorders, suicidal ideation, and psychosis)^4–19^. Furthermore, research suggests that genetic risk for PTSD covaries with trauma type, timing, severity, and degree of exposure^22–28^. Critically, research suggests that the dissociative subtype of PTSD and informally recognized subtypes of major depressive disorder (MDD; e.g., anxious depression, and psychotic subtype) may develop as a result of childhood trauma history, are characterized by distinct neurobiological mechanisms, and require different treatment approaches^29–34^. This suggests that common neurobiological factors among trauma-exposed individuals may contribute to heterogeneity in psychiatric symptoms and treatment responsivity; neurobiological phenotypes of trauma-induced psychiatric dysfunction transcend traditional diagnostic categories. In order to understand observed variability in the long-term effects of trauma exposure, it may be necessary to examine the relationship between variability in the nature and extent of trauma exposure and associated trauma-induced neurobiological alterations.

Research suggests that individual differences in the expression and activity of serotonin 1-A receptor (5-HT1A) are associated with a multitude of psychiatric symptoms commonly seen in trauma-exposed individuals^35–40^. Previous reviews have synthesized literature related to the role of 5-HT1A in depression, anxiety, memory, fear learning, impulsivity, suicide, and social dysfunction^35–40^. In addition, a multitude of preclinical studies have examined trauma-induced alterations in 5-HT1A^41^. Though the preclinical literature is consistent in suggesting that trauma alters 5-HT1A expression, specific findings diverge; some studies have observed increasing expression^41^, while others have observed the opposite^42^. In addition, many studies have presented conflicting evidence regarding specific brain regions in which increases and decreases occur^41–43^. Here, we synthesize the literature, including numerous preclinical studies, examining differences in alterations in 5-HT1A expression following trauma exposure. We suggest that observed inconsistencies may be accounted for in part by the influence of differences in the type of trauma subjects are exposed to. Furthermore, we suggest that the same is likely true of other molecular targets and that consideration of trauma type is a key aspect of accurate data interpretation. Thus, we examine the literature on 5-HT1A as an exemplar of a more general observation: in order to understand the neurobiology of trauma, one must account for covariance of trauma type and neurobiological alterations.

## 5-HT1A

The 5-HT1A receptor is one of seven inhibitory G protein-coupled serotonin receptors and is encoded by the HTR1A gene^44^. It is found in high density in areas associated with serotonin release (raphe nuclei), memory (hippocampus), fear (amygdala), pleasure (septum), and higher-order cognition (cerebral cortex)^38,45–51^. Physiologically, 5-HT1A activity in areas of high density as well as in several areas of lower density regulates neurotransmitter (e.g., dopamine, acetylcholine, noradrenaline, GABA, and glutamate) and hormone (e.g., cortisol and oxytocin) release as well as neural activity and functional connectivity^52–58^. In addition, 5-HT1A activity has been shown to modulate a wide array of adaptive and maladaptive behaviors. For example, in rodents, the 5-HT1A agonist 8-OH-DPAT has been found to increase impulsive action at low doses, and decrease impulsive action at higher doses^59^. Moreover, both agonists and antagonists of 5-HT1A have been shown to have antidepressant effects in preclinical models^60,61^. The region-specific nature of the physiological effects of 5-HT1A may be relevant to its impact on adaptive and maladaptive behaviors.

Among trauma-exposed individuals, numerous human studies have identified social behaviors as key differentiating factors which separate resilience from vulnerability (e.g., Refs. ^62,63^). Though it is generally accepted that 5-HT1A exerts strong effects on social behavior, those effects are highly complex^64,65^. For example, one study found that the 5-HT1A full agonist 8-OH-DPAT, the 5-HT1A partial agonist ipsapirone, and the region-specific partial agonist of 5-HT1A autoreceptors/antagonist of 5-HT1A heteroreceptors MDL-73,005-EF each led to a distinct pattern of effects on social behavior in rodents^64^. Regarding the overall frequency of pro-social behavior, 8-OH-DPAT led to an increase, MDL-73,005-EF led to a decrease, and ipsapirone had no effect^64^. In addition to the overall effects, each of the three drugs led to a specific combination of increases and decreases in each of six specific pro-social behaviors, six specific aggressive behaviors, and five specific defensive behaviors^64^. In addition, one study found that treating female marmoset monkeys with the 5-HT1A agonist 8-OH-DPAT impaired pair bond quality and decreased female sexual behavior^66^. By contrast, flibanserin, a regionally selective postsynaptic 5-HT1A heteroreceptors agonist/5-HT2A antagonist, improved pair bond quality and increased female sexual behavior^66^. Studies such as these have demonstrated the ability of different types of alterations in 5-HT1A activity (as well as its interactions with other receptors) to affect behaviors (both adaptive and maladaptive) shown to develop in the wake of trauma exposure.

In addition to social functioning, 5-HT1A modulates numerous trauma-relevant behaviors. A multitude of preclinical and human studies have linked 5-HT1A expression and activation to a number of well-known trauma sequalae; examples include depression (for reviews, see Refs. ^35,44,67,68^), anxiety (for reviews, see Refs. ^36,68^), memory (for reviews, see Refs. ^38,69–71^), fear learning (for review, see Ref. ^37^), impulsivity (for review, see Ref. ^39^), suicide (for review, see Ref. ^40^), substance abuse (e.g., Ref. ^72^), social dysfunction (for review, see Ref. ^64^), and pharmacological treatment responsivity (for reviews, see Refs. ^73,74^). Given the variety of trauma-related behaviors and neurobiological substrates that 5-HT1A receptors modulate, it is reasonable to hypothesize that heterogeneity in trauma-induced 5-HT1A alterations contributes to heterogeneity in psychiatric symptom expression. However, while numerous reviews discuss the impact of 5-HT1A on behaviors which are implicated in trauma, no works of which we are aware have synthesized the literature regarding trauma’s impact on 5-HT1A.

We argue that the importance of synthesizing the literature regarding the relationship between trauma exposure and 5-HT1A expression is that 5-HT1A can act as an exemplar of a general principle: that trauma type, chronicity, and duration likely have an impact on many (and possibly all) trauma-induced molecular alterations. Many of the fundamental mechanisms through which stress alters gene expression in general (e.g., transcription factors, miRNA, etc.) and 5-HT1A expression specifically (e.g., Freud-1 and miR-135a) regulate the expression of multiple genes rather than just one particular gene^44^. Thus, it is unlikely that different types of trauma or degrees of exposure would differentially impact the expression of one gene but not others. Though any number of molecular targets could theoretically be used to demonstrate this principle, 5-HT1A was used in this article for both practical and theory-driven reasons.

Our justification for selecting 5-HT1A as an exemplar here is twofold. First, because 5-HT1A is among the most widely studied molecular mechanisms of stressor-related psychopathology, a robust 5-HT1A focused preclinical literature exists^44^. This is critical because examination of the importance of trauma type and extent of exposure requires comparison of studies for which all other methodologies overlap; only a small portion of the literature meets all of those criteria. Second, by focusing on a molecular target that is widely studied in relation to stressor-related disorders but not often studied in relation to trauma, this work aims to call attention to a possible gap in the literature. In addition to PTSD, we believe that this work has the potential to inform the literature on depression, anxiety, and many other psychiatric diagnoses linked with 5-HT1A dysfunction.

## 5-HT1A and trauma: human studies

To date, very few published studies have directly examined the association between trauma exposure and 5-HT1A expression in humans. In addition, none have accounted for trauma type. However, a recent positron emission tomography (PET) study observed a negative correlation between chronic psychosocial stress and 5-HT1A availability in vivo in the hippocampus, anterior cingulate cortex, and insular cortex in humans^75^. Furthermore, using PET, subjects with PTSD have been found to have elevated 5-HT1A availability across 13 brain regions^76^. Notably, the results of some PET studies have been shown to vary as a function of which outcome measure researchers employ. For example, when researchers examining the relationship between PTSD and 5-HT1A availability used binding potential nondisplaceable uptake (BP_ND_; the outcome measure employed in the psychosocial stress study) rather than binding potential free plasma concentration (BP_F_), no relationship between PTSD and 5-HT1A was observed^76,77^. Another issue that limits interpretation of these findings is the cross-sectional nature of the study designs. Without longitudinal data it is not possible to determine whether dysregulated 5-HT1A expression occurred as a result of stress exposure (traumatic or otherwise) or represents a preexisting risk factor for stressor-induced disorders. For example, elevated 5-HT1A availability has been observed in subjects with remitted MDD and has been found to be a heritable preexisting risk factor for MDD^78,79^. While no published human studies have used longitudinal designs to study the effect of trauma on 5-HT1A expression, a body of preclinical literature suggests that trauma exposure may alter 5-HT1A expression.

## Preclinical models of traumatic stress exposure

In both humans and rodents, the line between non-trauma stress exposure and traumatic stress exposure is often blurry and imprecisely defined. In fact, the “Diagnostic and Statistical Manual” (DSM) definition of trauma exposure has been altered multiple times^80^. Though each DSM has presented a binary definition of PTE exposure, it is unclear whether the present dichotomous approach is adequate^80^. As mentioned previously, qualitatively different types of traumas can produce distinct neurobiological and behavioral consequences (e.g., Ref. ^21^). In addition, while the DSM uses a binary approach to define the boundary between “stress” and “trauma,” it may be important to focus more on qualitative aspects of distinct types of intense stressors and less on a dichotomous variable. For example, both criterion-A-type and noncriterion-A-type childhood maltreatment have been found to be negatively correlated with fractional anisotropy in the inferior longitudinal fasciculus^81^. Controversies regarding the use of a binary definition of trauma, which may incorrectly exclude some extreme stressors while incorrectly amalgamating others, have led to calls for a more fine-grained approach^80^.

The ambiguous and heterogeneous nature of PTEs is reflected in the preclinical trauma literature. A number of different preclinical models have been used to study trauma, including single prolonged stress (SPS), predator threat, restraint stress (RS; aka immobilization stress), inescapable electric shock (IES), fear conditioning (FC), chronic unpredictable stress (CUS; aka chronic variable stress), chronic social defeat (CSD), maternal separation (MS), and MS unpredictable stress. These paradigms and their relationship with trauma have been described and reviewed elsewhere (e.g., Refs. ^53,82–85^). Of note, CUS is an umbrella term encompassing any paradigm which involves daily exposure to one or more different stressors in a randomized sequence for 1 week or longer^86^. In this work, we only include CUS paradigms that include exposure to at least one of the aforementioned acute traumatic stressors. Just as many of the DSM-defined criterion A traumas were not included in the original definition of trauma exposure^80^, most of these preclinical trauma models were not initially conceptualized as representing trauma (e.g., RS, IES, FC, CUS, CSD, and MS)^82,83^. Due to the multifaceted and heterogeneous nature of potentially traumatic events, previous reviews have stated that no single preclinical model of trauma can adequately capture all aspects of trauma and that an ideal approach should draw from a variety of distinct models^82,83^.

While not traditionally viewed as a trauma paradigm, forced swim (FS) has been described as an uncontrollable and anxiogenic life-threatening situation^87^ and has been used in several studies to model trauma-related abnormalities in neurobiology, memory, and pain^88–90^. The behavioral effects of both acute and chronic FS stress have been measured using a number of additional preclinical tests (e.g., sucrose preference test, tail suspension test, elevated plus maze, open field test, social exploration test, Morris water maze, object location test, prepulse inhibition, cocaine preference, ethanol preference) (e.g., Refs. ^91–110^). Based on those tests, numerous studies have found evidence that FS can induce preclinical dysfunctions reflective of trauma sequalae including anhedonia (e.g., Ref. ^92^), anxiety (e.g., Ref. ^110^), social anxiety (e.g., Ref. ^100^), cognitive dysfunction (e.g., Ref. ^108^), depression (e.g., Ref. ^93^), and substance abuse (e.g., Ref. ^109^). Based on the intense nature of the stressor and its capacity for causing behavioral dysfunctions reflective of multiple facets of trauma-induced psychopathology, FS may represent an additional model of potentially traumatic event exposure. Thus, in order to provide a comprehensive picture of covariance of trauma type and 5-HT1A alterations, FS is included in this review.

Importantly, preclinical trauma models vary qualitatively in ways that make them appropriate analogs of different types of traumatic event exposure. In humans, interpersonal traumas (e.g., physical assault, sexual assault, and family violence) are qualitatively different from non-interpersonal traumas (e.g., severe accident, natural disaster, and life-threatening illness); this contributes to differences in psychiatric symptom severity and outcomes^111,112^. Similarly, some preclinical models incorporate a clear social component (e.g., CSD and MS), while others lack a social component (e.g., FC and RS)^83^. Additional clinically relevant qualitative factors which vary among preclinical trauma models include degree of threat of bodily harm, actual physical pain inflicted, and predictability^83^. In addition to qualitative differences, preclinical trauma models can vary in duration (time per exposure) and chronicity (days of exposure)^83^. In humans, increases in both duration and chronicity of trauma exposure, or number of lifetime exposures to trauma increase subjective peri-traumatic suffering and have been linked to increased symptom severity and qualitatively different psychiatric outcomes^112–118^. Evidence suggests that both qualitative and quantitative differences in rodent trauma models contribute to differences in their impact on rodent behavior and neurobiology^83^. Thus, differences in neurobiological response to various preclinical models of trauma may provide insight into the role of trauma type, duration, and chronicity in contributing to neurobiological heterogeneity among individuals with psychiatric disorders.

## Types of traumatic stress exposure and 5-HT1A alterations

As discussed above, different preclinical paradigms can be seen as analogs of distinct types of traumatic events; preclinical results suggest that when controlling for all other factors, exposure to qualitatively different traumatic events (preclinical paradigms) differentially affects alterations in 5-HT1A mRNA expression. For example, preclinical studies have found that exposure to CUS (approximates chronic exposure to various unpredictable and uncontrollable traumas) led to a decrease in 5-HT1A mRNA expression in all hippocampal subfields compared to controls, but exposure to SPS (approximates single exposure to prolonged perceived life threat) led to a decrease in hippocampal 5-HT1A mRNA expression that was restricted to the CA1 and dentate gyrus^42,119^. In one study, chronic exposure to 20 min of FS (approximates chronic and predictable exposure to perceived threat of drowning) did not alter 5-HT1A mRNA expression in any hippocampal region^119^. Comparison of these findings reveals that animals exposed to different preclinical paradigms, and thus qualitatively different traumatic events, experienced substantially different alterations in 5-HT1A mRNA expression; trauma-induced alterations in hippocampal 5-HT1A mRNA expression may be affected by both quality and frequency of trauma exposure.

Though alterations in mRNA expression represent transcriptional alterations in gene expression, posttranscriptional mechanisms may also cause changes in gene expression, especially during dynamic processes such as those which occur after trauma exposure^120^. Posttranscriptional mechanisms include any process after the transcription of DNA into mRNA, which affect subsequent steps of gene expression^120^. Exposure to SPS, CF + SPS (SPS preceded by five days of FC), or CUS have led to concurrent increases in 5-HT1A mRNA expression and 5-HT1A expression^41,119–122^. The concurrent nature of these changes in 5-HT1A mRNA and 5-HT1A protein expression suggests that transcriptional mechanisms are at least partially responsible for the observed change in 5-HT1A expression in these studies. However, two studies found that exposure to RS (approximates prolonged and uncontrollable confinement and threat of bodily harm) or FS (approximates perceived threat of drowning) led to incongruities in 5-HT1A mRNA expression and 5-HT1A expression^123,124^. For example, one study found that exposure to chronic RS led to an increase in 5-HT1A mRNA expression, but a decrease in 5-HT1A expression in the prefrontal cortex^123^. The opposing direction of alterations in 5-HT1A mRNA expression and 5-HT1A expression suggests that posttranscriptional mechanisms led to the decrease in 5-HT1A expression. Furthermore, when controlling for all other factors, one study found that RS and FS exposure led to opposite changes in 5-HT1A antagonist binding through posttranscriptional mechanisms; RS led to decreases (CA3 and dentate gyrus), while FS led to increases (CA2 and cortex), though neither exposure affected 5-HT1A mRNA expression^124^. Based on these findings, the degree to which trauma exposure impacts 5-HT1A expression through transcriptional versus posttranscriptional mechanisms may vary by trauma type. Furthermore, when trauma exposure does impact 5-HT1A expression through posttranscriptional mechanisms, the direction of change and region affected may also depend upon trauma type. Taken as a whole, this evidence suggests that the impact of trauma exposure on 5-HT1A expression, including the region, direction, and mechanism of change, is influenced by and potentially dependent in part on the type of trauma an individual is exposure to.

Further supporting our proposal that different trauma types may alter 5-HT1A through different mechanisms, research suggests that alterations in the 5-HT1A repressor Freud-1 may play a role in altering 5-HT1A expression after some, but not all traumas. In one study, exposure to RS led to reduced Freud-1 mRNA and protein in the prefrontal cortex as well as the expected concurrent increase in prefrontal 5-HT1A mRNA in Sprague-Dawley rats^123^. In another study, exposure to CSD (approximates chronic exposure to interpersonal violence) led to decreased prefrontal 5-HT1A mRNA expression in Wistar rats without affecting Freud-1 mRNA expression^125^. Of note, researchers also used different strains of rats (Sprague-Dawley rats and Wistar rats), which may have contributed to observed findings. However, one study which used only one breed of rat examined the effect of nontraumatic stressors in the prefrontal cortex and found that each of four qualitatively different types of stress led to distinct alterations in both mRNA and protein levels of 5-HT1A, Freud-1, and NUDR (NUDR acts as a 5-HT1A autoreceptor repressor and 5-HT1A heteroreceptor enhancer)^126^. Overall, this evidence suggests that different types of stress, including different types of traumatic stress, can alter 5-HT1A expression through different mechanisms.

Preclinical pharmacological studies provide additional supporting evidence for our thesis that different types of traumas differentially affect 5-HT1A. Different types of trauma may differentially alter the physiological impact of 5-HT1A activity. One study found that pretreatment with the 5-HT1A agonist ipsapirone differentially affected increases in extracellular levels of adrenocorticotropic releasing hormone, corticosterone, and plasma renin concentration induced by RS, FS, and FC (approximates exposure to predictable physical harm)^127^. In addition, one study found that the 5-HT1A agonist 8-OH-DPAT attenuated the corticosterone response to FC but not RS or IES (approximates exposure to inescapable physical harm)^128^. Based on these results, the observed differential impact of trauma type on 5-HT1A alterations likely has downstream physiological consequences including altered neuro-endocrine functioning.

Though they examine different rodent strains and therefore do not include a direct comparison to other trauma types, additional studies suggest that specific trauma-induced 5-HT1A alterations may have specific behavioral consequences. For example, one study found that, in male Sprague-Dawley rats, SPS led to an increase in 5-HT1A expression in the hippocampal CA1 region as well as impaired spatial memory performance^129^. Local CA1 injection of the 5-HT1A agonist 8-OH-DPAT further exacerbated the SPS-induced impairment, suggesting an impairing effect of SPS-induced 5-HT1A upregulation in the CA1 on spatial memory^129^. By contrast, decreased 5-HT1A expression and activity in the prelimbic (PrL) cortex may be a critical mechanism of CSD-induced anxiety. One study found that, in female mandarin voles, CSD led to decreased 5-HT1A expression in the PrL, decreased PrL serotonin levels, and increased anxious behavior^130^. Local PrL injection of 8-OH-DPAT reversed the CSD-induced anxiety in exposed voles, while local PrL injection of WAY-100635 (a 5-HT1A antagonist) caused anxious behaviors in control voles^130^. Of note, in CSD-exposed voles, 5-HT1A autoreceptor expression was increased in the dorsal raphe nucleus (which projects to the PrL), suggesting that the decreased PrL serotonin levels were caused by a region-specific increase in 5-HT1A expression^130^. Overall, these results suggest that region-specific trauma-induced increases and decreases in 5-HT1A expression and activity may have impairing behavioral effects. Given that different types of trauma lead to different region-specific alterations in 5-HT1A, this suggests that the differences in behavioral impact observed in different trauma types may be related to the observed differences in 5-HT1A alterations.

## Chronicity and duration of traumatic stress exposure and 5-HT1A alterations

As is the case with different types of traumas, preclinical results suggest that, when controlling for all other factors, differences in duration (time per exposure) or chronicity (days of exposure) lead to different alterations in 5-HT1A expression and 5-HT1A mRNA expression. For example, in one study, exposure to 30 min of FS led to an increase in 5-HT1A agonist binding and 5-HT1A antagonist binding in the hippocampus and cortex^124^. By contrast, exposure to only 15 min of FS did not alter 5-HT1A agonist binding or 5-HT1A antagonist binding in the hippocampus or cortex^124^. Similarly, findings from two studies suggest that exposure to 14 days of CUS led to a decrease in hippocampal 5-HT1A mRNA expression, but exposure to 7 days of the same CUS paradigm did not alter hippocampal 5-HT1A mRNA expression^42,119^. As the extent of trauma exposure leads to different clinical consequence in humans, preclinical evidence suggests that both duration and chronicity of exposure appear to impact 5-HT1A expression.

Differences in chronicity of trauma exposure can differentially impact 5-HT1A expression in complex ways. For example, in one study, exposure to CSD led to a transient increase in 5-HT1A binding in the claustrum, which was found when exposure lasted 2 days, but not when exposure lasted 10 or more days^43^. However, exposure to the same CSD paradigm led to a stable decrease in 5-HT1A binding in the posterior cingulate, which was found when exposure lasted 10, 21, or 28 days^43^. Exposure for 28 days also led to decreases in the parietal cortex, prefrontal cortex, regio retrobulbaris, and CA1 hippocampal region, which were not found when exposure lasted <28 days^43^. These results support a possible region-specific dose-response relationship between chronicity of trauma exposure and 5-HT1A binding. Interestingly, no change was detected in the raphe for any chronicity of CSD up to 28 days^43^. In another study, 2 h of exposure to RS led to an increase in hippocampal 5-HT1A binding when exposure lasted 1 day and a further increase in 5-HT1A binding when exposure lasted 5 days^131^. In contrast with the overall increase in hippocampal 5-HT1A binding observed between 1 and 5 days of exposure, increases in the CA4 subregion were transient; increases in 5-HT1A binding in the CA4 were observed when exposure lasted for 1 day but not when it lasted for 5 days^131^. Overall, findings suggest that changes in length of exposure may lead to distinct region-specific changes in the degree and direction of the relationship between trauma exposure and 5-HT1A expression; these changes can be transient in nature or delayed in onset. Taken together, preclinical findings support the assertion that differences in qualitative and quantitative aspects of trauma exposure contribute to variation in subsequent alterations in 5-HT1A expression.

## Clinical/pharmacological implications

The exact role of 5-HT1A in the etiology of trauma-related psychiatric disorders is unclear. However, the prevailing consensus is that 5-HT1A is influential in the development of depression, anxiety, fear, and memory impairment^35–37^. In addition, one study finds that individuals with PTSD may have elevated 5-HT1A availability^76^. Further elucidation of 5-HT1A’s behavioral impact, including the degree to which it varies as a function of trauma history, may lead to the development of highly targeted treatments, and betterment of outcomes through personalized treatment^44,132^. For example, preclinical studies suggest that biased 5-HT1A agonists, which specifically target autoreceptor or heteroreceptor signaling, may be more effective in treating depression and anxiety than currently available 5-HT1A agonists^133–135^. In addition, recent literature suggests that drugs capable of acting on specific mechanisms of 5-HT1A expression could lead to major breakthroughs in treating depression^44^. However, targeting the correct mechanism for alteration of 5-HT1A may be critical^44,136^. Given the impact of trauma type and extent of exposure on 5-HT1A, we theorize that trauma history may be a crucial determinant of response to future breakthrough treatments.

In support of our theory, previous correlational studies and randomized controlled trials have found evidence to suggest that individuals with the same diagnosis but different trauma histories respond differently to currently available pharmacological treatments^137–144^. Thus, individuals with different trauma histories may require different pharmacological treatments, likely in part due to the differential effects of those trauma histories on gene expression. Failure to account for trauma history during pharmacological studies may obscure trauma-induced heterogeneity in responsivity, thus concealing a drug’s potential efficacy (or lack thereof) in individuals with particular trauma histories and delaying progress in clinical practice. Based on this evidence, in order to achieve personalized treatment, one must first account for the differential impact of different types of trauma and degrees of exposure.

## 5-HT1A as an exemplar

Though the primary focus of this review has been 5-HT1A, the implications of these findings likely extend to additional molecular targets. Multiple preclinical studies suggest that different types of trauma and degrees of exposure can lead to alterations in molecular targets other than 5-HT1A. For example, exposure to CUS led to multiple region-specific increases and decreases in dopamine D1-like receptor binding, but exposure to RS did not impact D1-like receptor binding in any of those regions^145^. Similarly, in the hippocampal CA2, exposure to RS led to an increase in serotonin-7 receptor mRNA expression but exposure to CUS did not^146^. Additional examples exist, including research focused on mu opioid and glucocorticoid receptors^147,148^. Similarly, multiple studies have found evidence to suggest that different types of trauma exposure and different degrees of trauma exposure differentially affect gene expression in humans^149–153^. For example, in one study, profiles of gene expression in peripheral blood cells which distinguished individuals with PTSD from controls were almost completely distinct (98% nonoverlapping), depending on whether subjects with PTSD had a history of childhood abuse exposure^153^. Importantly, in addition to PTSD, the impact of trauma type and degree of exposure on gene expression has been evident in individuals with MDD and borderline personality disorder^150^. Taken together, these studies provide direct evidence for the importance of trauma type and degree of exposure in modulating the effect of trauma on genetic expression, which extends beyond 5-HT1A and beyond PTSD.

Previously, we synthesized evidence to suggest that different types of traumas can alter 5-HT1A through different mechanisms, including the 5-HT1A repressor Freud-1. A closer examination of specific mechanisms of 5-HT1A alterations provides a useful paradigm for understanding the complexity of the concurrent impact of different trauma types and degrees of exposure on many molecular mechanisms. For example, the 5-HT1A repressor Freud-1 also represses the dopamine receptor D_2_^154^. Thus, differences in the impact of various types of trauma on Freud-1 likely lead to differences in expression of not only 5-HT1A, but also D_2_ and any other mechanisms affected by Freud-1. In addition, we provided evidence that different types of stressors differentially impact the dual-effect 5-HT1A autoreceptor repressor and 5-HT1A heteroreceptor enhancer NUDR. In addition to its effect on 5-HT1A, NUDR also regulates transcription of Proenkephalin^155^. Notably, many transcriptional and posttranscriptional mechanisms of 5-HT1A expression also affect multiple molecular targets^44^. For example, the 5-HT1A autoreceptor enhancer Pet-1 also plays a role in activating expression of the nicotinic acetylcholine receptor^156,157^. In addition, 5-HT1A promotors MAZ and Sp1 decrease NMDA receptor subunit type 1 promoter activity^158,159^. Furthermore, in addition to posttranscriptional regulation of 5-HT1A, miR-135a also regulates the serotonin transporter, PHLPP2, and FOXO1^44^. These findings illustrate the general principal that the mechanisms underlying differential impacts of trauma type and chronicity of exposure on 5-HT1A expression likely lead to differential impacts on other molecular targets as well. Furthermore, they provide insight into the fact that traumatic stressors may regulate a wide array of molecular targets through diffusely acting mechanisms.

## Limitations and future directions

This paper does not provide an exhaustive review of the literature on 5-HT1A and trauma. Many studies which found alterations in 5-HT1A expression induced by rodent trauma models were excluded from mention in this work due to a lack of direct comparability arising from methodological discrepancies^58,160–184^. Future preclinical studies can further elucidate the impact of differences in trauma type and extent of exposure on changes in the expression of 5-HT1A and other molecular targets by consciously and deliberately accounting for trauma type. One particularly noteworthy gap in the current literature concerns early life trauma. To date, models of early life trauma have been associated with increases^183^, decreases^174^, or no change^184^ in 5-HT1A expression. However, methodological differences (e.g., rodent strain, rodent sex, chronicity of exposure, additional experimental stressors, time since trauma exposure at sacrifice, and index of 5-HT1A expression) preclude direct comparisons between trauma models or between different developmental periods as they relate to 5-HT1A expression. Future studies should endeavor to resolve this issue.

More studies are needed in order to identify relationships between specific categories of trauma and resultant alterations in gene expression. In addition, these findings have yet to be extended to humans. Only one published study in humans has evaluated the relationship between 5-HT1A and trauma exposure, with null results^185^. Of note, however, trauma type was not controlled for and authors acknowledged that the study may have lacked sufficient power to detect an association^185^. Future studies should examine the association between trauma and 5-HT1A expression in humans, specifically with respect to differences in trauma type and chronicity of exposure. Given the known adverse impacts of aversive childhood experiences and the divergence in genetic expression associated with childhood and adult trauma, differences in developmental timing of trauma should also be considered.

While it seems clear based on the reviewed preclinical literature that trauma type and chronicity of exposure impact gene expression, no available preclinical studies examining 5-HT1A distinguish behaviorally affected and resilient individuals. In the preclinical literature, the importance of separating behaviorally affected animals from resilient animals is becoming increasingly clear^82,83^. Studies have found that some post-trauma alterations in gene expression that are present in the group average may only apply to affected animals, while others may only apply to resilient animals^82,83^. Thus, with the exception of studies that measure the behavioral effects of 5-HT1A agonists or antagonists in addition to measuring gene expression, most currently available studies do not provide information to distinguish adaptive alterations in 5-HT1A from maladaptive alterations. Future studies should apply methodology such as cut-off behavioral criteria and behavioral profiling in order to distinguish alterations in 5-HT1A that are seen in resilient animals from those seen in affected animals. Of note, cut-off behavioral criteria and behavioral profiling are applied analytically to postexposure behavioral data and do not necessarily require a change in study design^82,83^. Thus, it may be possible to apply these techniques to archival data. Though some research questions may require the design of new studies in order to incorporate specific behavioral effects of interest, we recommend applying cut-off behavioral criteria and/or behavioral profiling to archival data wherever feasible. In addition to being highly practical, the use of archival data is in line with ethical best practice as it does not cause any additional suffering to animals^186^.

This review focuses on the importance of considering differences in the nature and chronicity of trauma exposure in neurobiological research. However, other key variables warrant similar consideration. Specifically, recent research has emphasized the importance of sex differences, risk factors, and individual differences in response to the same trauma^82^. We propose that these two approaches need not be mutually exclusive. For example, in rodents, qualitatively different types of nontraumatic stressors have been found to lead to sex-dependent differential alterations in mRNA and density of 5-HT1A and specific 5-HT1A transcription factors^126^. Future studies should examine the degree to which the interaction of stressor type and sex extends to rodent trauma models and human trauma exposure. This approach could also be extended to other relevant risk factors, such as inherited genetic differences, which are shown to alter trauma’s impact on 5-HT1A expression as well as behavior^182^. Specific single nucleotide polymorphisms alter the ability of specific mechanisms to impact gene expression^187^. Specific mechanisms underlying the relationship between trauma and gene expression depend on trauma type and level of exposure. Therefore, future studies should investigate specific interactions between genes, sex, and trauma type as they relate to 5-HT1A expression.

## Conclusions

We have presented preclinical evidence supporting our contention that differences in the type and chronicity of exposure to trauma leads to differences in the region-specific posttraumatic alterations in 5-HT1A. Furthermore, differences in the nature of trauma and extent of exposure to trauma appear to lead to differences in the mechanisms underlying changes in 5-HT1A expression; even when two different traumas lead to the same directional change in 5-HT1A expression in a given region, it is possible that different mechanisms are driving the change. Furthermore, this phenomenon does not appear to be specific to 5-HT1A and evidence suggests that it may translate to humans. Based on the evidence presented in this review, future studies aimed at understanding the differential molecular alterations that are generally associated with certain categories of trauma exposure may eventually inform a more targeted approach to pharmacological treatments for trauma-exposed individuals. In order to understand the neurobiology of trauma, it is crucial to consider both trauma type and extent of exposure.
